# Rapidly progressive respiratory failure with multiple halo signs on computed tomography in a patient with primary cardiac angiosarcoma derived from the right atrium: a case report

**DOI:** 10.1186/s12890-020-01366-6

**Published:** 2020-12-09

**Authors:** Masaoki Saito, Takeshi Saraya, Miku Oda, Toshinori Minamishima, Ken Kongoji, Aya Isomura, Masachika Fujiwara, Kyoko Soejima, Haruyuki Ishii

**Affiliations:** 1 Department of Respiratory Medicine, Kyorin University School of Medicine, 6-20-2 Shinkawa, Mitaka City, Tokyo 181-8611 Japan; 2 Department of Cardiology, Kyorin University School of Medicine, Shinkawa, Mitaka City, Tokyo 181-8611 Japan; 3 Department of Pathology, Kyorin University School of Medicine, Shinkawa, Mitaka City, Tokyo 181-8611 Japan

**Keywords:** Primary cardiac angiosarcoma, Halo sign, Endomyocardial biopsy

## Abstract

**Background:**

Primary cardiac neoplasms are extremely rare, with an autopsy incidence of 0.0001–0.003%. Primary cardiac sarcoma is usually derived from the right atrium and it manifests as chest pain, arrhythmia, hemoptysis, dyspnea, and fatigue. The most common target organ for metastasis of primary angiosarcoma is the lungs, but the radiological-pathological correlation has been rarely reported.

**Case presentation:**

A 38-year-old healthy Japanese man was admitted to our hospital with persistent hemoptysis, exaggerated dyspnea, and two episodes of loss of consciousness in the past 3 months. Non-enhanced thoracic computed tomography (CT) revealed multiple scattered nodules with halo signs. Contrast-enhanced thoracic CT revealed a filling defect in the right atrium, which corresponded to the inhomogeneously enhancing tumor in the right atrium on enhanced electrocardiogram-gated cardiac CT. On day 2, acute respiratory failure occurred, and the patient was placed on mechanical ventilation. The patient was diagnosed with primary cardiac angiosarcoma based on the urgent transcatheter biopsied specimen of the right atrium mass and was treated with intravenous administration of doxorubicin. However, his respiratory status rapidly deteriorated, and he died on day 20. Postmortem biopsy showed that the multiple lung nodules with the halo signs corresponded to the intratumoral hemorrhagic necrosis and peripheral parenchymal hemorrhage in their background, suggesting the fragility of the lung tissue where the tumor had invaded, which caused hemoptysis. Furthermore, two episodes of loss of consciousness occurred probably due to a decreased cardiac output because of a massive tumor occupying the right atrium, recognized as an inhomogeneous centripetal enhancement on enhanced electrocardiogram-gated cardiac CT.

**Conclusions:**

This case clearly demonstrated that primary cardiac angiosarcoma could expand in the right atrial cavity, which led to a decreased cardiac output resulting in repeated syncope, together with the fragility of lung tissue by tumor invasion, thereby generating a halo sign on thoracic CT.

## Background

Primary cardiac neoplasms are extremely rare, with an autopsy incidence of 0.0001–0.003% [1]. Approximately, 90% cases of primary cardiac angiosarcoma occur in the right atrium, while less than 5% occur in the left atrium or ventricles. Lung metastasis from angiosarcoma derived from the right atrium often demonstrates a halo sign on thoracic CT. However, few reports have described the pathological analysis of halo signs from the perspective of the fragility of the lung tissue where the tumor invaded, which caused hemoptysis. Herein, we report a case of primary angiosarcoma with multiple halo signs on thoracic CT, successfully diagnosed using transcatheter endomyocardial biopsy with pathological analysis of halo signs.

## Case presentation

**Fig. 1:**
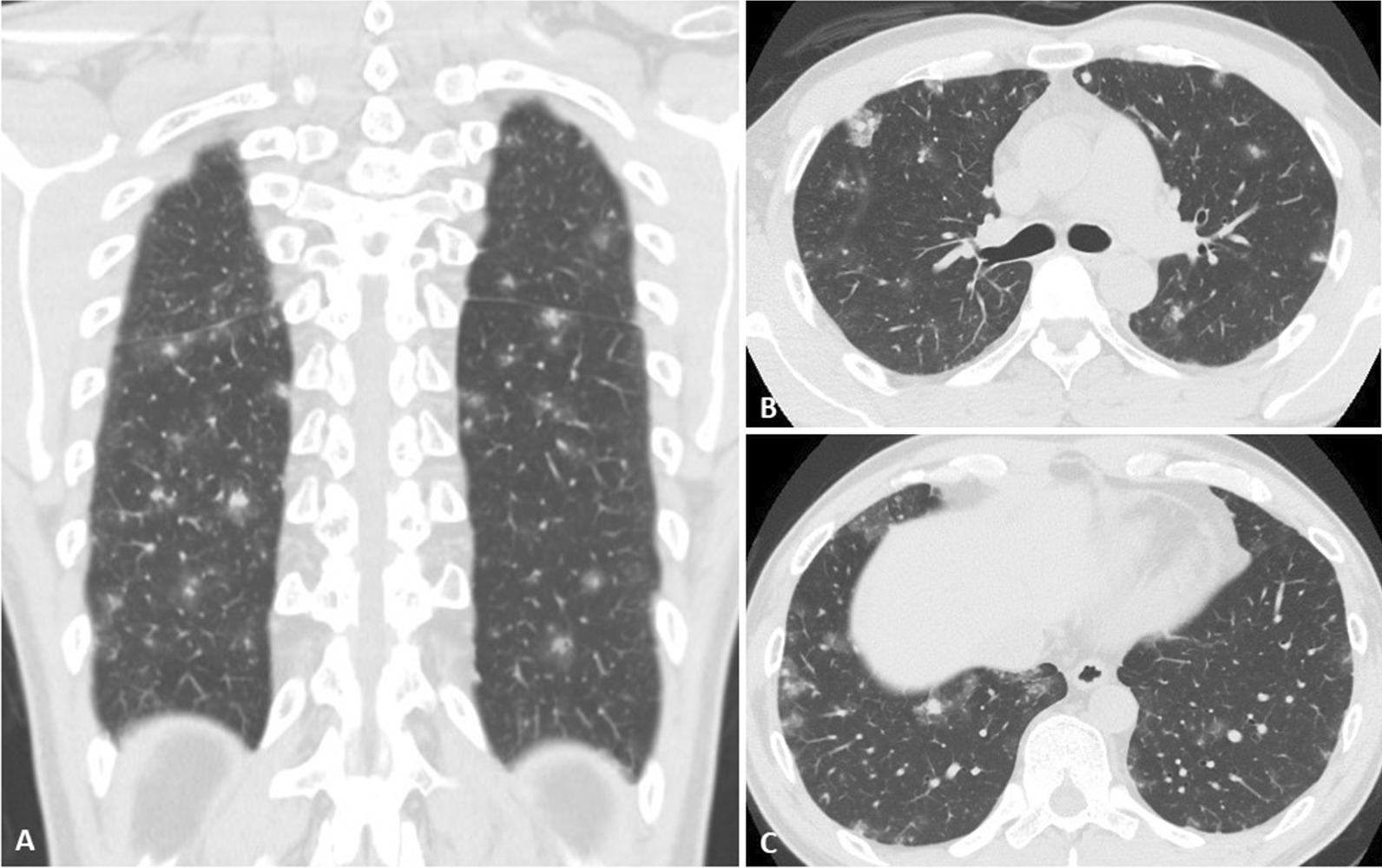
Thoracic computed tomography (CT) scan. **a** Coronal view of thoracic CT performed 2 months before admission demonstrates multiple scattered nodules with ground-glass opacities, the so-called “halo sign.” **b**, **c** Axial views of thoracic CT demonstrates “halo signs” located on the outer side of the lung, suggesting hematological spread

A 38-year-old healthy Japanese man from China was admitted to our hospital (day 1); thoracic computed tomography (CT) revealed abnormal lung lesions. The patient was a former smoker (two pack-years) with no history of illicit drugs use or dust exposure. Three months before admission, he had two episodes of loss of consciousness in China; however, echocardiography and head CT showed no abnormal findings. Two months previously, he noticed hemoptysis and experienced dyspnea. Immediately after he returned to Japan, thoracic CT (Fig. 1a, coronal view) evaluation was performed at a local hospital that demonstrated multiple scattered nodules, most of which were surrounded by ground-glass opacities (GGO), the so-called “halo signs,” which were located on the outer side of the lungs (Fig. 1b, c), suggesting hematological spread.

The patient’s vital signs were as follows: blood pressure, 108/60 mmHg; body temperature, 36 °C; respiratory rate, 18 breaths/min; tachycardia (heart rate, 108 beats/min); and hypoxemia (blood oxygen saturation using pulse oximetry of 89% on ambient air). Physical examination revealed conjunctival rim pallor and coarse crackles in the early to mid-inspiratory phase in both the lung fields.

**Fig. 2:**
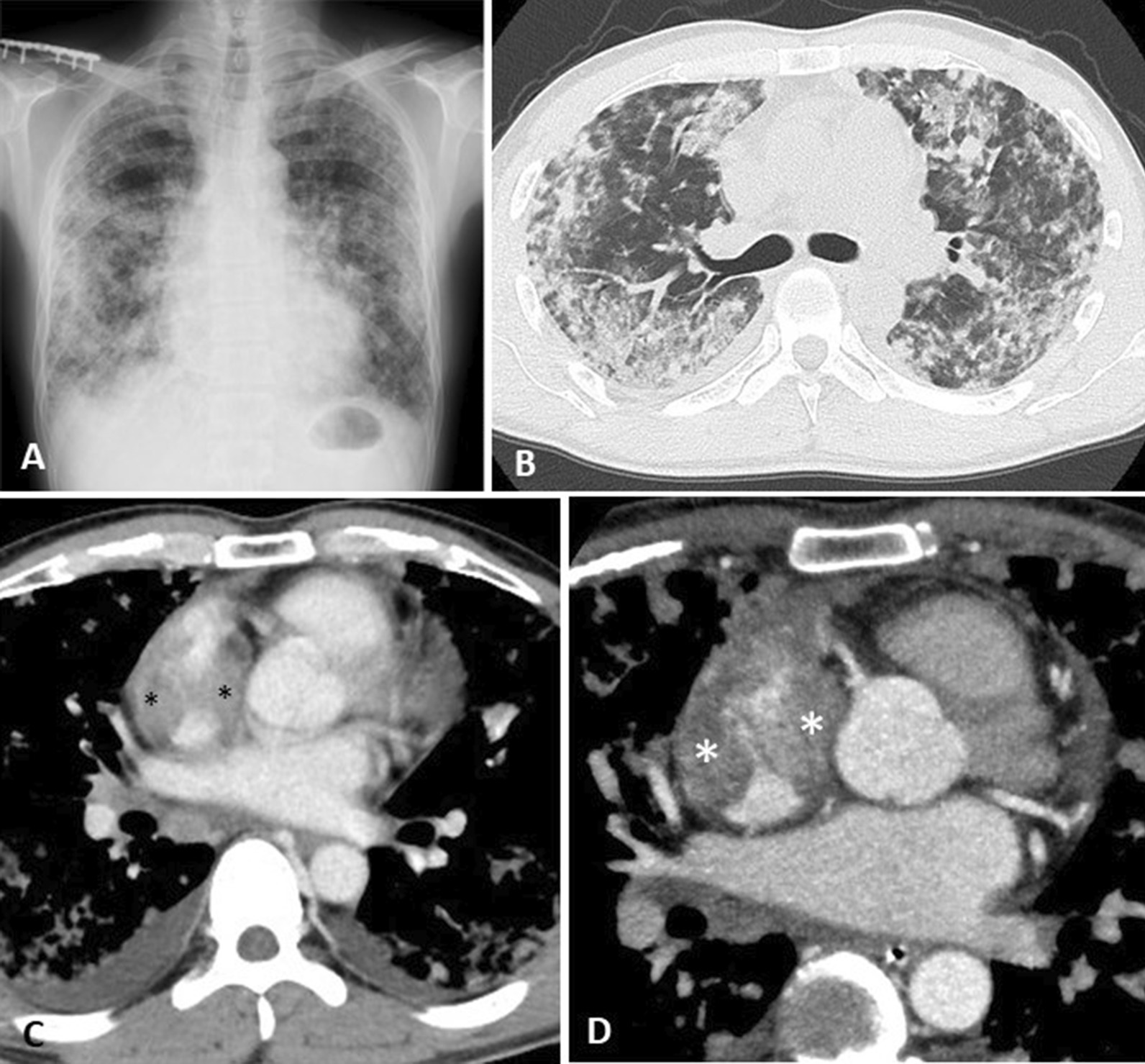
Chest radiograph and thoracic computed tomography (CT) on admission. **a** Chest radiograph on admission shows non-segmental bilateral lung infiltrations, predominantly in the right-sided middle to lower lung fields. **b** Non-enhanced thoracic CT shows non-segmental consolidation with ground-glass opacities or multiple scattered nodules in both the lung lobes. **c** Contrast-enhanced thoracic CT reveals a filling defect in the right atrium (black asterisks). **d** Enhanced electrocardiogram-gated cardiac CT reveals an inhomogeneously enhancing tumor in the right atrium (white asterisks)

Serum laboratory data showed severe anemia with a hemoglobin level of 5.6 g/dL, mild or moderate elevation in white blood cell counts of 11,700/µL, and C-reactive protein level of 10.2 mg/dL. On admission, chest radiograph (Fig. 2a) showed non-segmental bilateral lung infiltration predominantly seen in the right-sided middle to lower lung fields. Non-enhanced thoracic CT simultaneously showed non-segmental consolidation in both the lung lobes with scattered multiple nodules (Fig. 2b), but no apparent lymphadenopathies were noted. Serum data for *Aspergillus galactomannan*, *Cryptococcus* antigen, and T-SPOT tests were negative. No pathogens were isolated from sputum. Further, myeloperoxidase anti-neutrophil cytoplasmic antibodies and serine proteinase 3-anti-neutrophil cytoplasmic antibodies were also negative. Furthermore, none of the serum tumor marker, such as carcinoembryonic antigen, sialyl Lewis X-i antigen, α-fetoprotein, or human chorionic gonadotropin, was elevated.

Careful examination of contrast-enhanced thoracic CT suggested a filling defect in the right atrium (Fig. 2c, black asterisks), which corresponded to the inhomogeneously enhancing tumor in the right atrium (Fig. 2d, white asterisks) on enhanced electrocardiogram-gated cardiac CT. On day 2, acute respiratory failure occurred and the patient was placed on mechanical ventilation.

**Fig. 3:**
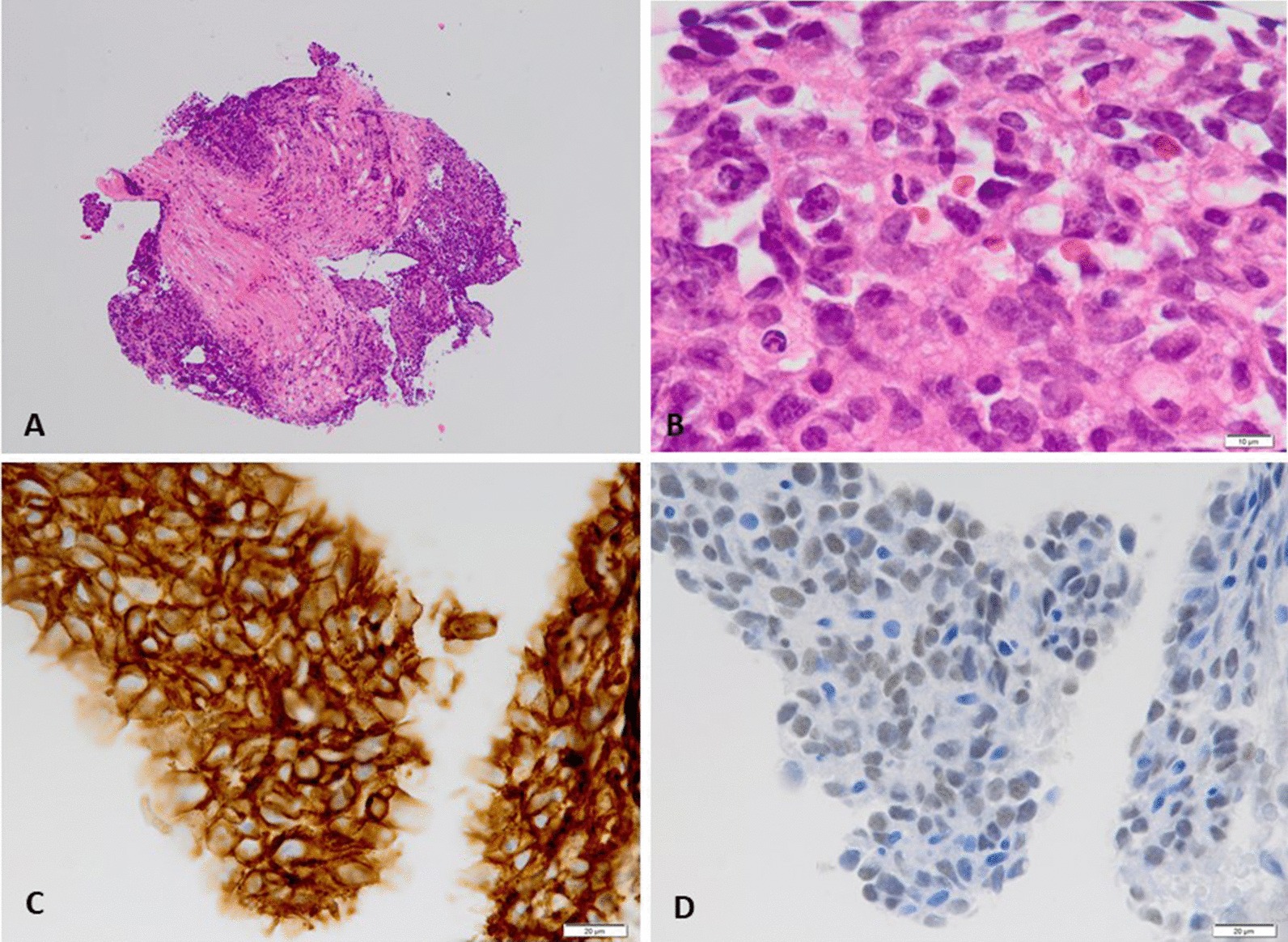
Transcatheter endomyocardial biopsy findings. **a** On hematoxylin–eosin staining, the transcatheter endomyocardial biopsied specimen demonstrates abundant atypical cells invading the myocardial tissue (40×). **b** Immunohistochemical analysis reveals atypical cells partly forming capillary-like spaces (600×). **c** Staining with CD-31 (400×). **d** Staining with ERG (400×)

On day 8, urgent transcatheter endomyocardial biopsy was performed, which revealed abundant atypical cells invading the myocardial tissue (Fig. 3a, 40×) on hematoxylin–eosin staining. These atypical cells partly formed capillary-like spaces (Fig. 3b, 600×) and were stained with FLI-1 (figure not shown), CD-31 (Fig. 3c, 400×), and ERG (Fig. 3d, 400×) on immunohistochemical analysis, suggesting an endothelial origin of the tumor.

**Fig. 4:**
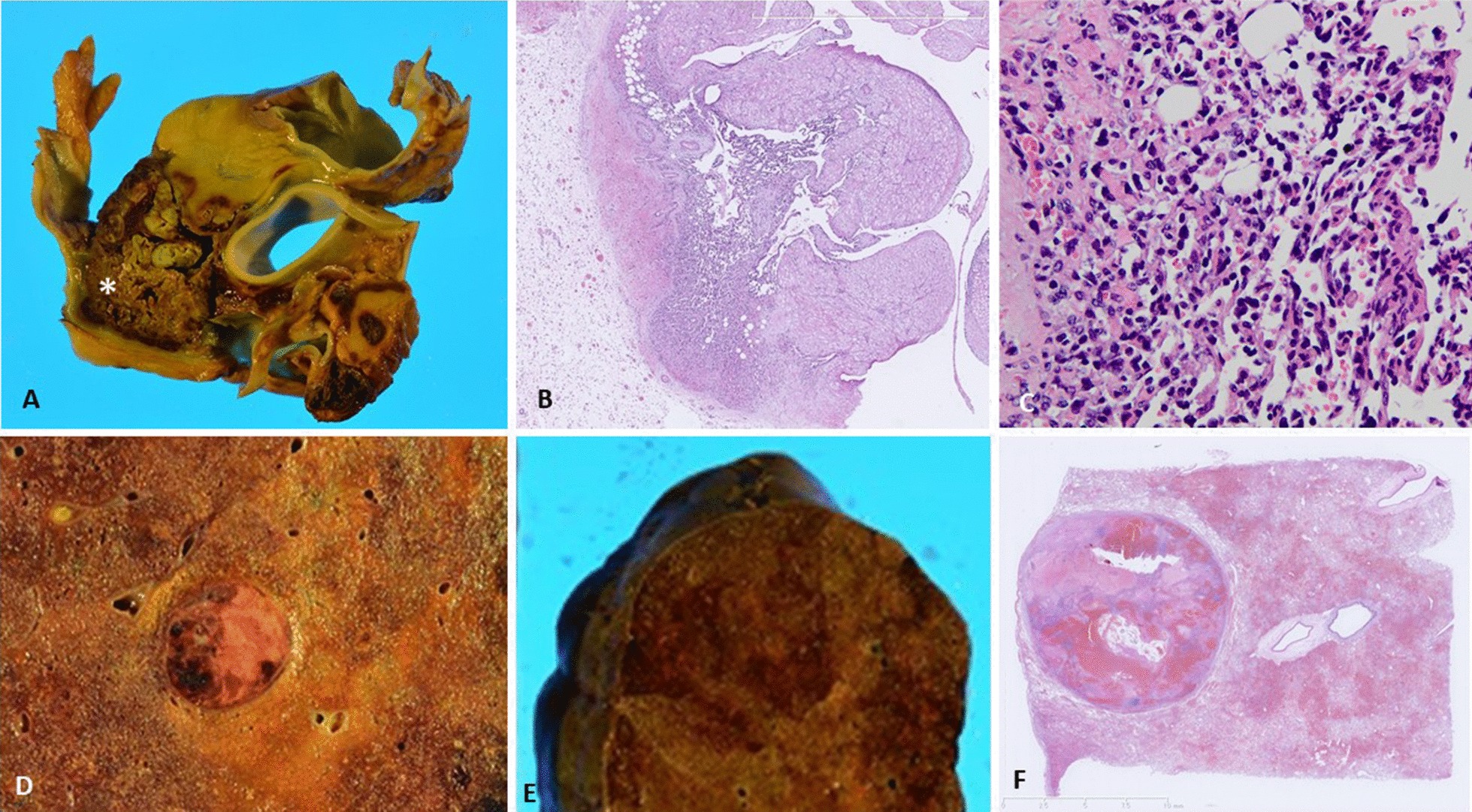
Autopsy findings. **a** Massive tumor occupying the right atrium (asterisk). **b** Tumor cells infiltrating the myocardium (20×). **c** Hematoxylin–eosin staining reveals atypical cells partly forming capillary-like spaces (200×). **d** The cut surfaces of the right lung reveals multiple nodules, measuring 1 cm in diameter. **e** A hemorrhagic infarct seen in the peripheral areas. **f** A loupe image of the glass slide of the lung shows a well-demarcated hemorrhagic tumor and severe breeding in the surrounding lung parenchyma

On day 10, based on the transcatheter biopsied specimen of the right atrium mass, the patient was tentatively diagnosed with primary cardiac angiosarcoma and treated with intravenous administration of doxorubicin (75 mg/m^2^). However, his respiratory status rapidly deteriorated, and he died on day 20. Postmortem autopsy demonstrated that the right atrium contained a massive tumor (Fig. 4a, asterisk), which also showed infiltrative growth in the myocardium (Fig. 4b, 20×). The tumor was composed of the similar atypical cells as seen in the transcatheter biopsied specimen (Fig. 4c, 200×). Cut surfaces of the right lung revealed multiple nodules, measuring 1 cm in diameter (Fig. 4d) together with a hemorrhagic infarct in peripheral areas (Fig. 4e). Histological features of the pulmonary nodules were identical to those of the cardiac tumor with accompanying intratumoral hemorrhagic necrosis and severe bleeding in the surrounding lung parenchyma (Fig. 4f). Hence, the patient was diagnosed with primary cardiac angiosarcoma derived from the right atrium with lung metastasis via hematological spread. The tumor also metastasized to the mediastinal and bilateral hilar lymph nodes and bilateral adrenal glands.

## Discussion and conclusions

Primary cardiac neoplasms are extremely rare [1]. Benign primary cardiac neoplasms, myxomas being the most frequent type, account for 75% of cases, whereas malignant ones, angiosarcomas, and rhabdomyosarcomas being the most common, account for 25% of cases [2]. Angiosarcoma is an aggressive, malignant endothelial-cell tumor of lymphatic or vascular origin accounting for 1–2% of all cases of sarcoma [3] and 30% of all cases of cardiac malignancy [2]. Approximately 90% cases of primary cardiac angiosarcoma occur in the right atrium, while less than 5% occur in the left atrium or ventricles [4]. Most patients are younger than 65 years of age and in the fourth decade of life. Furthermore, the disease shows a male predominance (male: female ratio = 2–3:1), as presented in this case.

With respect to clinical findings, primary cardiac angiosarcoma usually manifests as chest pain, arrhythmia, hemoptysis, dyspnea, and fatigue [5]; the latter three symptoms were noted in our case. Our patient had two episodes of loss of consciousness, probably due to a decreased cardiac output because of a massive tumor occupying the right atrium, which was seen as an inhomogeneous centripetal enhancement on enhanced electrocardiogram-gated cardiac CT.

This case clearly demonstrated a radiological-pathological correlation, the multiple lung nodules with the halo signs corresponded to the intratumoral hemorrhagic necrosis and peripheral parenchymal hemorrhage in the background, suggesting the fragility of the lung tissue where the tumor invaded, which caused hemoptysis.

The most common target organ for metastasis of primary angiosarcoma is the lungs, but the liver, lymph nodes, bone, adrenal glands, and spleen may also be involved; however, some reports of radiological findings of primary cardiac angiosarcoma with lung metastasis have been published [3, 6–8]. Based on these reports, peculiar thoracic CT findings of lung involvement in cardiac angiosarcoma showed different sized multiple nodules and multiple thin-walled cysts with or without GGO, ranging between 0.5–2.0 and 0.8–7.1 cm, respectively [6].

Hemorrhagic pulmonary nodules have a characteristic CT appearance, consisting of a central area of soft tissue attenuation surrounded by a halo of ground-glass attenuation. This halo sign has diverse differential diagnoses, such as hemorrhagic infectious processes, including invasive aspergillosis, candidiasis, coccidioidomycosis, cytomegalovirus, herpes simplex virus, and noninfectious causes, including Wegener granulomatosis, metastatic angiosarcoma, and Kaposi sarcoma. In this regard, this case had typical halo signs on thoracic CT, which rapidly progressed to severe GGO/consolidations in the next 2 months, suggesting massive infiltration of tumor cells.

However, the respiratory condition under mechanical ventilation cannot easily perform invasive qualitative diagnostic procedures, such as CT-guided biopsy, bronchoscopy, and surgery. Furthermore, Adem et al. [9] reported the clinical limitation of transbronchial lung biopsy for the diagnosis of metastatic angiosarcoma of the lung because of fragility of the tissue presenting as diffuse pulmonary hemorrhage. The diagnostic yield of endomyocardial biopsy was as low as 50% in a previous report [10]. Therefore, it has been used less frequently to diagnose cardiac tumors, as in the previous two reports [11, 12], even in the setting of non-mechanical ventilation.

In this regard, to the best of our knowledge, this is the first case of primary cardiac angiosarcoma successfully diagnosed using transcatheter endomyocardial biopsy in a patient on mechanical ventilation.

In conclusion, our patient had a rare cardiac tumor, but showed peculiar findings on careful consideration, such as multiple halo signs, hemoptysis, dyspnea, and repeated syncope, because of decreased venous return or tumor embolization derived from cardiac angiosarcoma in the right atrium.

## Data Availability

Not applicable.

## Abbreviations

CT: Computed tomography
GGO: Ground-glass opacities
